# Upper limb joint kinematics using wearable magnetic and inertial measurement units: an anatomical calibration procedure based on bony landmark identification

**DOI:** 10.1038/s41598-019-50759-z

**Published:** 2019-10-08

**Authors:** Pietro Picerno, Pietro Caliandro, Chiara Iacovelli, Chiara Simbolotti, Michele Crabolu, Danilo Pani, Giuseppe Vannozzi, Giuseppe Reale, Paolo Maria Rossini, Luca Padua, Andrea Cereatti

**Affiliations:** 1 School of Sport and Exercise Sciences, “e-Campus” University, Novedrate, Italia; 2 Unità Operativa Complessa di Neurologia, Fondazione Policlinico Universitario A. Gemelli IRCCS, Roma, Italia; 3 IRCCS Fondazione Don Carlo Gnocchi, Milano, Italia; 4 Department of Electrical and Electronic Engineering, University of Cagliari, Cagliari, Italia; 5 Department of Informatics, Bioengineering, Robotics and System Engineering, University of Genoa, Genova, Italia; 6 Department of Movement, Human and Health Sciences, University of Rome “Foro Italico”, Roma, Italia; 7 Dipartimento di Scienze dell’invecchiamento, Neurologiche, Ortopediche e della Testa-Collo, Università Cattolica del Sacro Cuore, Roma, Italia; 8 University of Sassari, Biomedical Sciences Department, Sassari, Italia

**Keywords:** Musculoskeletal models, Biomedical engineering

## Abstract

The estimate of a consistent and clinically meaningful joint kinematics using wearable inertial and magnetic sensors requires a sensor-to-segment coordinate system calibration. State-of-the-art calibration procedures for the upper limb are based on functional movements and/or pre-determined postures, which are difficult to implement in subjects that have impaired mobility or are bedridden in acute units. The aim of this study was to develop and validate an alternative calibration procedure based on the direct identification of palpable anatomical landmarks (ALs) for an inertial and magnetic sensor-based upper limb movement analysis protocol. The proposed calibration procedure provides an estimate of three-dimensional shoulder/elbow angular kinematics and the linear trajectory of the wrist according to the standards proposed by the International Society of Biomechanics. The validity of the method was assessed against a camera-based optoelectronic system during uniaxial joint rotations and a reach-to-grasp task. Joint angular kinematics was found as characterised by a low-biased range of motion (<−2.6°), a low root mean square deviation (RMSD) (<4.4°) and a high waveform similarity coefficient (R^2^ > 0.995) with respect to the gold standard. Except for the cranio–caudal direction, the linear trajectory of the wrist was characterised by a low-biased range of motion (<11 mm) together with a low RMSD (8 mm) and high waveform similarity (R^2^ > 0.968). The proposed method enabled the estimation of reliable joint kinematics without requiring any active involvement of the patient during the calibration procedure, complying with the metrological standards and requirements of clinical movement analysis.

## Introduction

Wearable magnetic and inertial measurement units (MIMUs), consisting of a three-axial accelerometer, gyroscope and a magnetometer, represent a self-contained alternative to conventional lab-based motion capture systems for joint angular kinematics assessment^1,2^. Their use is particularly favourable when motion analysis must be performed outside the laboratory (such as clinical settings) or for a long period of time^3^. Angular velocity, gravity and magnetic field vectors are used by specific sensor fusion algorithms to estimate the three-dimensional (3D) orientation of MIMUs with respect to a global coordinate system^4^. As joint kinematics is defined as the relative orientation of two adjacent bony segments^5,6^, the use of MIMUs for the estimate of a consistent and clinically sound joint kinematics requires knowledge of the orientation of the MIMU-embedded coordinate system with respect to the anatomical coordinate system of the body segment on which the MIMU is strapped. This information is retrieved during a sensor-to-segment axes calibration procedure (simply called “anatomical calibration”), which can be implemented in different ways. With special reference to upper limb kinematics, thorax, upper arm and forearm anatomical coordinate systems are generally determined according to one of the following approaches proposed in the literature: (a) matching the MIMU coordinate system with the anatomical coordinate system through manual alignment of the MIMU case^7^; (b) asking the subject to assume a given body segment configuration (“N-pose” or “T-pose”)^8^; (c) exploiting the direction of the angular velocity vector as measured during specific monoaxial rotations of a body segment together with the direction of the gravity vector as measured while keeping a segment’s axis aligned with the vertical line^9^. While simple manual alignment of the MIMU case could be critical from a repeatability perspective, in cases of severe impaired mobility, applying the second and third approaches could be difficult as the subjects may be not able to perform functional calibration tasks or assume specific postures. In addition, all three approaches may be particularly critical with bedridden acute patients as they are usually equipped with medical devices and apparatus the positioning of which might jeopardise the alignment of the MIMU with the underlying bone and limit the joint range of motion.

An alternative to overcome the aforementioned limitations is to determine the direction of the anatomical axes on the base of the bone morphology, particularly, from the position of a few selected palpable anatomical landmarks (ALs). To the best of the authors’ knowledge, a similar approach has been previously used for the estimation of lower limb joint angular kinematics^10^ and scapula orientation tracking^11^.

The aim of this study was to develop a MIMU-based anatomical calibration procedure to estimate linear and angular kinematics of an upper limb kinematic model and assess the procedure’s validity against a camera-based optoelectronic system.

## Material and Methods

### Anatomical calibration

Let ^**g**^**R**_**s**_ be a 3 × 3 orientation matrix describing the 3D orientation of the MIMU coordinate system *s* with respect to a common global coordinate system *g*:

$${}^{g}\,{{\bf{R}}}_{s}=[{}^{g}\,{{\bf{u}}}_{{x}_{s}},\,{}^{g}\,{{\bf{u}}}_{{y}_{s}},\,{}^{g}\,{{\bf{u}}}_{{z}_{s}}],$$

where the unit vectors **u** are the column elements of the matrix denoting the orientation of each single axis of *s* with respect to *g*.

**Figure 1:**
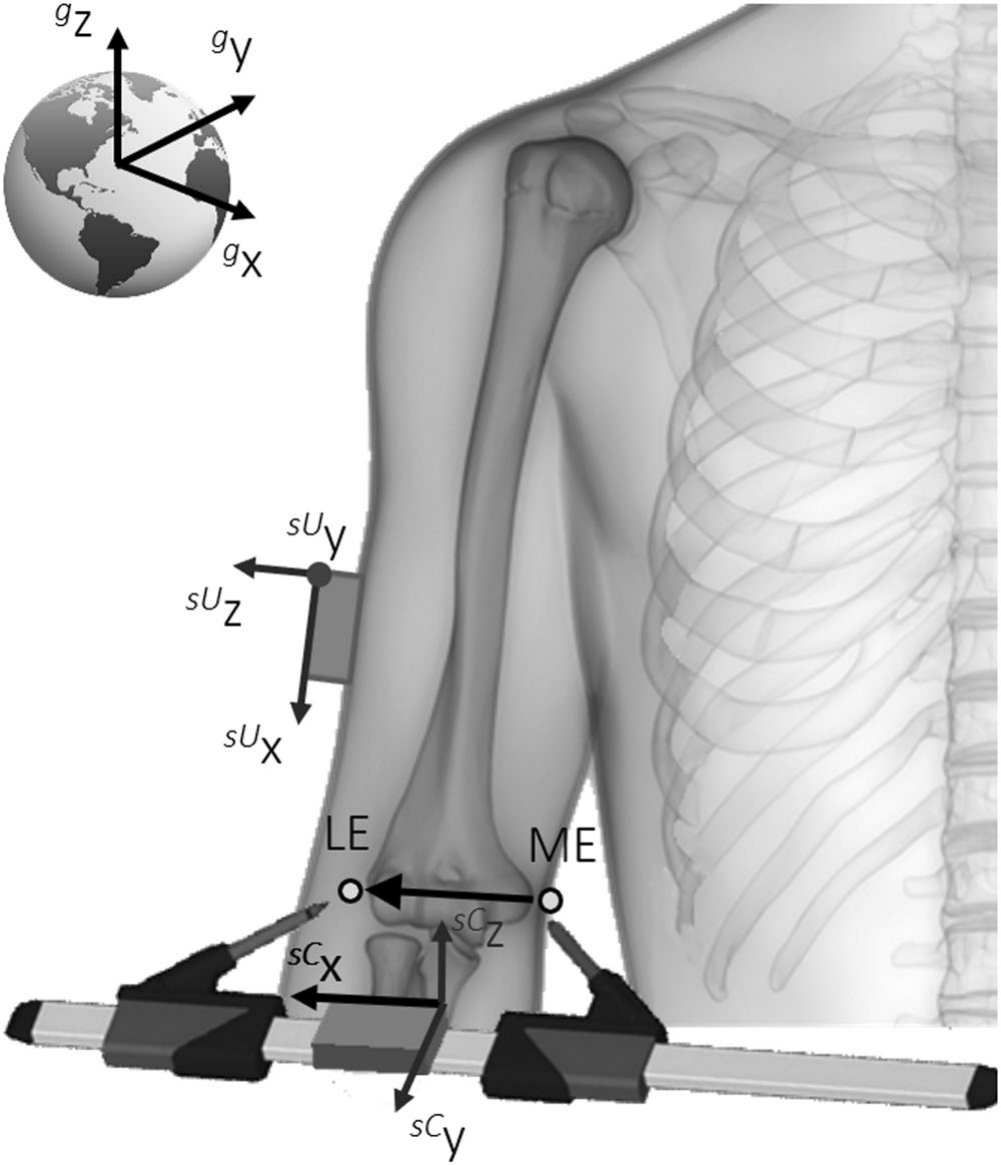
Anatomical calibration of a magnetic and inertial measurement unit (MIMU) fixed on a body segment. The direction of an anatomical axis as defined by two palpable ALs is measured in the MIMU’s global coordinate system using an extra MIMU hosted on a calliper-like device to point to the two considered anatomical landmarks. As the two MIMUs share the same global reference frame, the direction of the anatomical axis can be expressed with respect to the sensor-embedded coordinate system of the MIMU fixed on the segment and used to collect motion data.

With reference to the example shown in Fig. 1, a MIMU is strapped on the subject’s upper arm (*sU*) for collecting movement data. An extra MIMU is mounted on a light-weight calliper-like pointing device (*sC*), made of aluminium and having adjustable length, such that its x-axis is aligned with the axis joining the tips of the calliper’s arms (${}^{g}\,{{\bf{u}}}_{{x}_{sC}}$). The orientation of the axis defined by two pointed ALs (in Fig. 1, the line joining the medial epicondyle, ME, to the lateral epicondyle, LE) with respect to *g* can be directly retrieved from the first column of the 3 × 3 direction cosine matrix output by the sensor:

$${}^{g}\,\widehat{ME\,LE}={}^{g}\,{{\bf{u}}}_{{x}_{sC}},$$

and subsequently expressed in the MIMU coordinate system (*sU*) used to track the motion of the segment through the rigid, time-invariant transformation^10^.

$${}^{sU}\,\widehat{ME\,LE}={}^{g}\,{{\bf{R}}}_{sU}{(0)}^{-1}\cdot {}^{g}\,\widehat{ME\,LE}$$

Note that ${}^{g}\,{{\bf{R}}}_{sU}(0)$ and ${}^{g}\,\widehat{ME\,LE}$ are simultaneously collected and the rigid transformation is allowed as the two MIMUs share the same global coordinate system. If the same procedure is applied for a second axis, an upper arm-embedded anatomical coordinate system (*aU*) can be defined and rigidly associated to the MIMU strapped to the segment using a time-invariant rotation matrix ${}^{sU}{{\bf{R}}}_{aU}(0)$.

### Upper limb kinematic model

**Figure 2:**
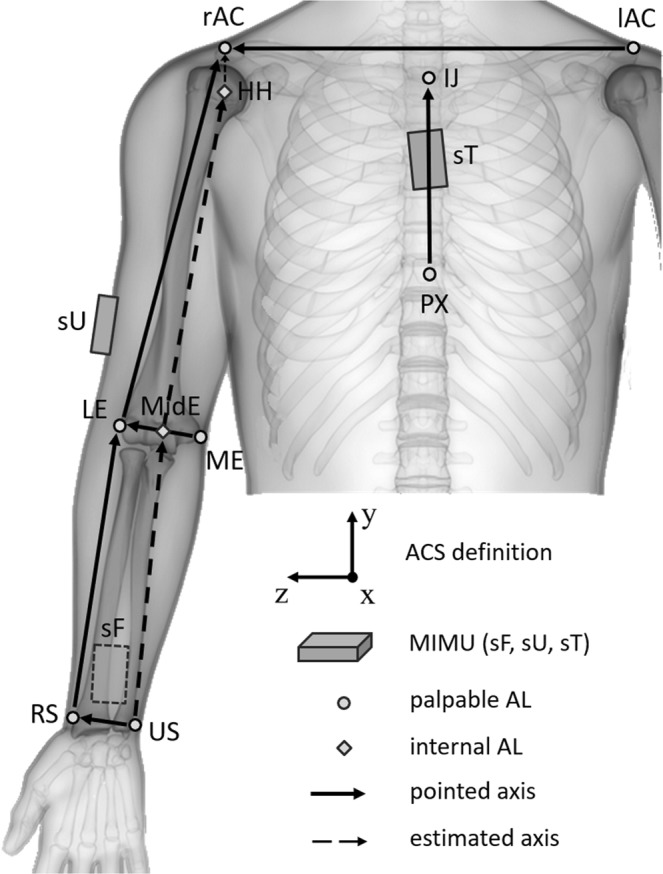
MIMU placement for the proposed upper body kinematic model and palpable anatomical landmarks (ALs) used for the anatomical calibration procedure. Note that the orientation of sensors on the thorax (sT), upper arm (sU) and forearm (sF) is arbitrary. Solid and dashed arrows represent the pointed and estimated anatomical axes, respectively. The former are calibrated, whereas the latter are determined using vector summation in Eq. 2 and Eq. 4 (MidE-US and HH-MidE, forearm and upper arm longitudinal axes, respectively) or estimated via anthropometrical modelling in Eq. 5 and Eq. 6 (rAC-HH). The anatomical coordinate system (ACS) definition for thorax, upper arm and forearm definition is also shown.

The adopted upper limb kinematic model comprises three rigid body segments: the thorax, humerus (upper arm), and radio-ulna (forearm) connected by spherical joints. Each body segment is associated to an anatomical coordinate system, the axes of which are determined according to the guidelines presented by the International Society of Biomechanics^12^ (see Eqs 1, 3 and 7 of the Appendix for details) and are based on the identification of the following palpable ALs: processus xiphoideus, incisura jugulars, right and left acromion, lateral and medial epicondyle, and the radius and ulnar styloid (Fig. 2). The pointing device also provides the inter-anatomical landmark distance along the axis defined by a pair of selected ALs. This information, in addition to the measured anatomical axes orientation and some anthropometric assumptions, is used to estimate the orientation of the upper arm and forearm longitudinal axes together with the distances between the shoulder and elbow joints centres (i.e. upper arm length) and between the elbow joint centre and ulnar styloid (i.e. forearm length; see Eqs. 2 and 4 of the Appendix for details). Figure 2 shows the MIMU setup for collecting the right-side upper limb kinematics together with the measured (pointed) and estimated (internal) anatomical axes used for the anatomical coordinate system determination.

#### Angular and linear joint kinematics

**Figure 3:**
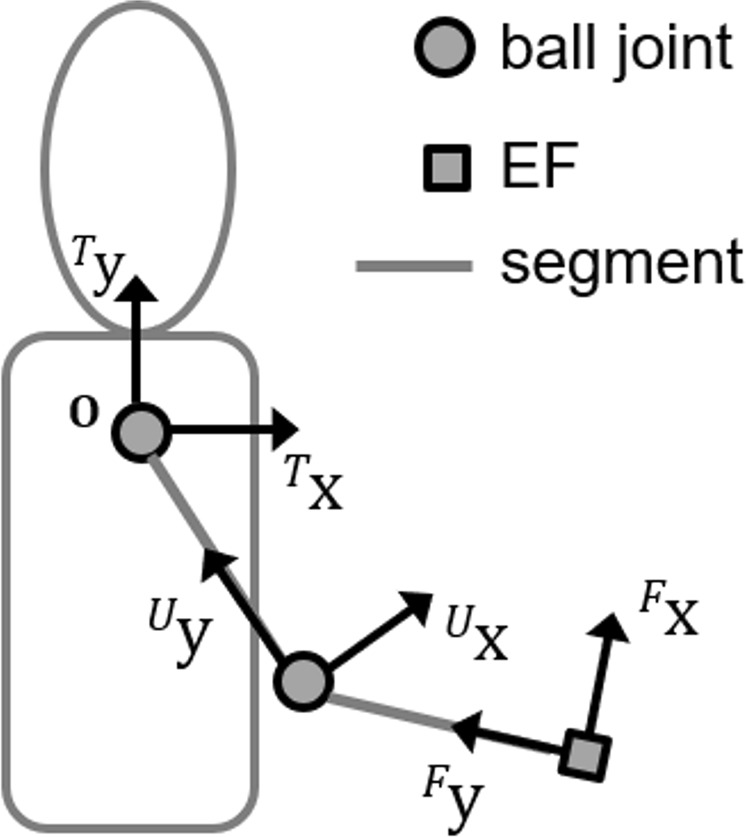
The two-link open kinematic model for the forward kinematics of the upper limb model. The position of the end-effector (i.e., the ulnar styloid) is retrieved with respect to the thorax anatomical frame, which origin is located in the shoulder (hinged, no translational degrees of freedom). Thorax, upper arm and forearm orientation are retrieved from the anatomically calibrated MIMUs placed on the relevant segments, while the length of the forearm and upper arm segments corresponds to the magnitude of the vectors estimated in Eq. 2 and Eq. 4 of the Appendix, respectively.

The orientation of the humerus was computed with respect to the thorax, whereas the orientation of the forearm was computed with respect to the humerus. The 3D shoulder and elbow joint angular kinematics were obtained by decomposing the relevant joint orientation matrices following the recommendations of the International Society of Biomechanics^12^. Furthermore, as the distances between shoulder and elbow joint centres and between the elbow joint centre and ulnar styloid are available in addition to the time-variant orientation of the upper arm and forearm anatomical coordinate systems with respect to the thorax, a homogeneous transformation matrix can be used to solve the forward kinematics of a two-link open kinematic chain (Fig. 3) at every sampled instant of time and obtain the instantaneous 3D position of the ulnar styloid (i.e. the end-effector of the chain) with respect to the thorax (see Eqs 8–10 of the Appendix)^13^. Table 1 summarises the model’s output.

**Table 1:** Summary of the output variables estimated by the MIMU-based upper limb kinematic model.

Model’s output	Joint	Acronym	Full name
Angular displacement	Shoulder	*α*_*h*_	plane of elevation
		*β*_*h*_	elevation
		*γ*_*h*_	axial rotation
	Elbow	*α*_*e*_	flexion-extension
		*β*_*e*_	prono-supination
Linear displacement	Wrist	[p_*x*_, p_*y*_, p_*z*_]	position of the ulnar styloid

### Experimental approach

The proposed anatomical calibration approach was validated using a six-camera optoelectronic (OPTO) stereophotogrammetric system (Smart-DX, BTS Bioengineering, Italy), considered the gold standard^14^, during two experimental sessions. In the first experimental session, we evaluated the accuracy of the proposed procedure in estimating shoulder and elbow 3D angular kinematics during monoaxial joint rotations. Fourteen young adults (four females and ten males, aged 34 ± 7 years) with no previous shoulder or elbow injury were enrolled in the first experiment to evaluate the performance of the method when wide angular rotations are considered.

A second experiment was performed to assess the accuracy of the 3D trajectory of the EF during a reach-to-grasp task that was chosen for its clinical relevance in the assessment of the residual motor function, particularly, in subjects with acquired brain injury^15^. The accuracy of the estimated 3D position of the EF was tested on six young-old subjects (four females and two males, aged 62 ± 13 years) with no previous shoulder or elbow injury and with a range of age similar to that of a typical acquired brain injury population. For both experiments, sample size was determined through an a priori power analysis on pilot data as detailed in the statistical analysis paragraph.

**Figure 4:**
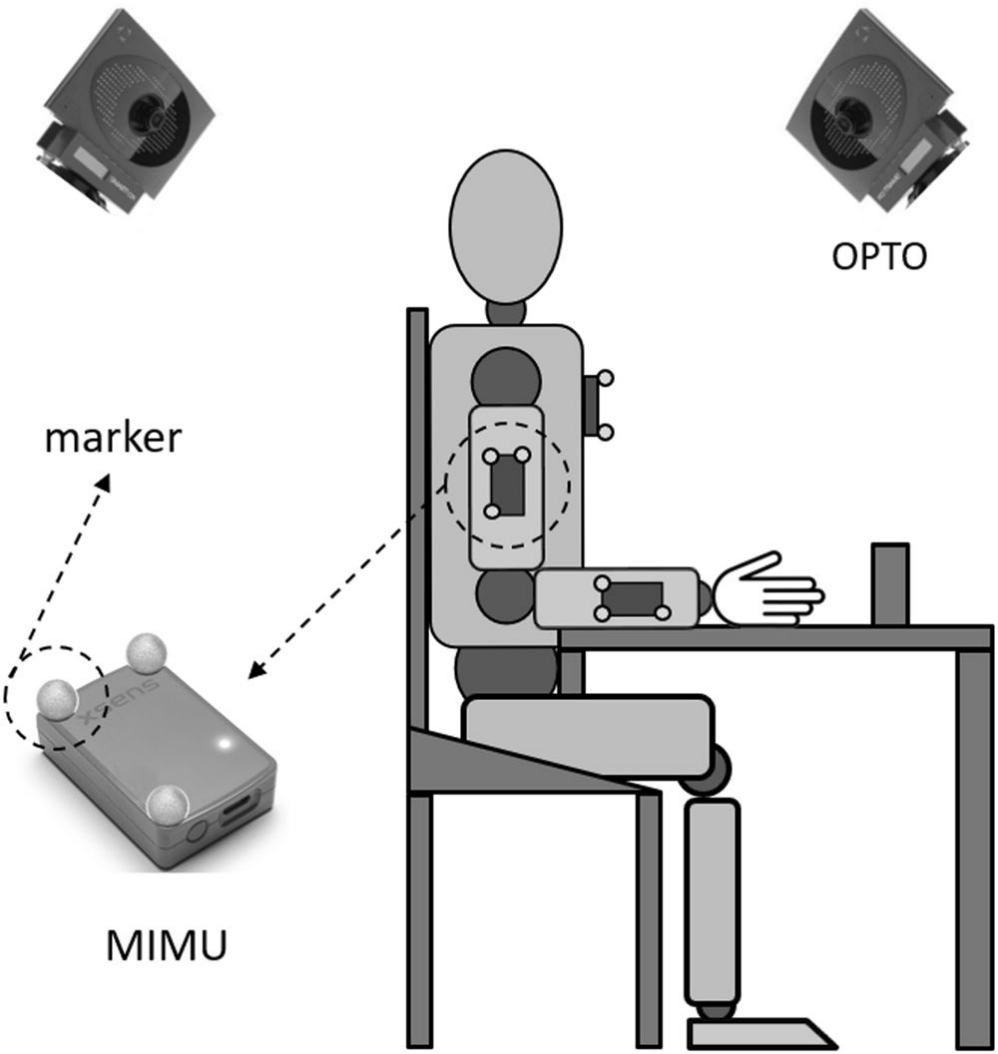
Measurement setup relative to the reach-to-grasp task showing both measurement systems employed in this study.

In both experiments, each participant was equipped with three MIMUs (MTw, Xsens Technologies BV) mounted on the thorax, upper arm and forearm, as depicted in Fig. 2. Three retro-reflective markers were attached on each MIMU for validation. Figure 4 shows an example of the measurement setup utilised in this study with both measurement systems used for collecting reference (OPTO) and MIMU-based data. Prior to the execution of the tasks, the full anatomical calibration procedure was performed by a single operator who, first, identified and marked the selected palpable ALs depicted in Fig. 2, and then used the calliper-like device to point the previously marked ALs and compute the relevant quantities in Eqs 1–4 and Eqs 6–7 of the Appendix. Following the latter procedure, additional retro-reflective markers were placed on the previously marked ALs to ensure that each anatomical coordinate system was consistently defined for both the MIMU-based and OPTO-based methods used to collect the 3D markers’ position. Markers located on ALs were removed after their position was recorded in a technical coordinate system defined by the three markers placed on the MIMUs during a static trial according to the CAST protocol^16^. The two measurement systems were electronically synchronised using an external trigger signal, and data were collected at a rate of 100 samples/s. MIMU orientation data were retrieved using the manufacturer’s proprietary software (MT Manager, v.4.6). A spot-check of MIMU performance was conducted at the experiment’s location prior to performing the tests to verify inter-MIMU global coordinate system consistency^17^. The study was approved by the Ethic Committee of the “Agostino Gemelli” University Polyclinic Foundation (Rome, Italy), and informed consent was signed by the participants. All experiments were performed in accordance with relevant guidelines and regulations.

#### Experiment A: Joint angular kinematics assessment

Starting from the anatomical position, subjects performed the test in the following order: (1) arm elevation in the sagittal plane, (2) arm elevation in the scapular plane, (3) arm elevation in the frontal plane, (4) elbow flexion-extension (6). Finally, with the elbow flexed at 90°, subjects performed: (5) shoulder external-internal rotation, and (6) elbow prono-supination. Three non-consecutive repetitions of each of the abovementioned motor tasks, performed at a self-selected speed, were recorded.

#### Experiment B: Wrist trajectory assessment during reach-to-grasp movements

Fifteen non-consecutive reach-to-grasp movements were performed from a seated position with the dominant arm. Seat and table heights were adjusted such that the seated position was comfortable with the subjects’ thighs parallel to the ground, feet flat on the ground and knees flexed at about 90°. Subjects started in a position with the hand on the table in neutral pronation, elbow flexed at approximately 90° and upper arm adducted along the trunk. They were instructed to reach forward at a self-selected speed, grab the object and return to their initial position. The object, centred with respect to the subject’s longitudinal axis, was placed on the table at a subject-specific distance corresponding to a quasi-complete extension of the elbow avoiding scapular protraction and elevation.

### Data reduction and statistical analysis

For the first experiment, the *β*_*h*_ range of motion (RoM) was computed during arm elevation in the sagittal plane, arm elevation in the scapular plane and arm elevation in the frontal plane, whereas *γ*_*h*_ RoM was computed during shoulder external–internal rotation, *α*_*e*_ RoM was computed during elbow flexion–extension and, finally, *β*_*e*_ RoM was computed during elbow prono–supination. Shoulder plane of elevation values (*α*_*h*_) were also computed during arm elevation in the sagittal, scapular and frontal planes. For the second experiment, the magnitude of the wrist position vector at the grasp point ($|\overrightarrow{{\bf{p}}}|$_*grasp*_) and the linear RoM of the wrist along the x, y and z directions were computed at every reach-to-grasp cycle and considered for statistical analysis.

For both experiments, absolute agreement between the two systems in the considered angular and linear variables was assessed through Bland–Altman analysis corrected for the effects of repeated measurements^18^. The presence of heteroscedasticity was checked by calculating the Kendall rank correlation coefficient τ^19^: when τ > 0.1 a logarithmic transformation of the data was assessed before calculating bias and 95% upper and lower limits of agreement^20,21^. Finally, the estimated (MIMU-based) and reference (OPTO-based) angular and linear trajectories were compared at each movement cycle in terms of root mean square deviation (RMSD) and via the “linear fit method” using typical parameters: *R*^2^ (waveform similarity), *a*_1_ (amplitude) and *a*_0_ (offset)^22^. In brief, *R*^2^ and *a*_1_ are coefficients that range from 0 to 1 (where 1 implies same shape and amplitude), whereas *a*_0_ quantifies the offset between the two curves when *a*_1_ tends to 1. When *R*^2^ > 0.5, the assumption of linearity is considered valid and *a*_1_ can be interpreted as meaningful^23^.

The normal distribution of data was assessed using the Shapiro–Wilk test. The alpha level of significance was set at 0.05. Sample size analysis (β = 0.2), based on Bland–Altman plots^24^, was performed on pilot data that included three subjects performing experiment A and one subject performing experiment B. For experiment A, power analysis was performed using *β*_*h*_ RoM relative to arm elevation in the sagittal plane and *α*_*e*_ RoM relative to elbow flexion–extension as these were considered the most involved degrees of freedom during the reach-to-grasp task. For experiment B, power analysis was performed using $|\overrightarrow{{\bf{p}}}|$_*grasp*_. For experiment A, the expected mean difference between methods was 0.616° for *β*_*h*_ RoM and 0.548° for *α*_*e*_ RoM, whereas the maximum allowed difference was set to 5° resulting in a minimum required number of pairs equal to 7 and 27 for *β*_*h*_ RoM and *α*_*e*_ RoM, respectively; however, the number of pairs eventually chosen for experiment A equalled 42 (14 subjects × 3 repetitions). For experiment B, the expected mean difference between methods was 7 mm and the maximum allowed difference was set to 20 mm resulting in a minimum required number of pairs equal to 62; however, the number of pairs eventually chosen for experiment B equalled 90 (6 subjects × 15 repetitions).

Signal processing and kinematic analysis was performed in MATLAB (The MathWorks, Inc., Natick, USA), and statistical analysis was performed using MedCalc Statistical Software v. 18.5 (MedCalc Software, Ostend, Belgium).

## Results

**Figure 5:**
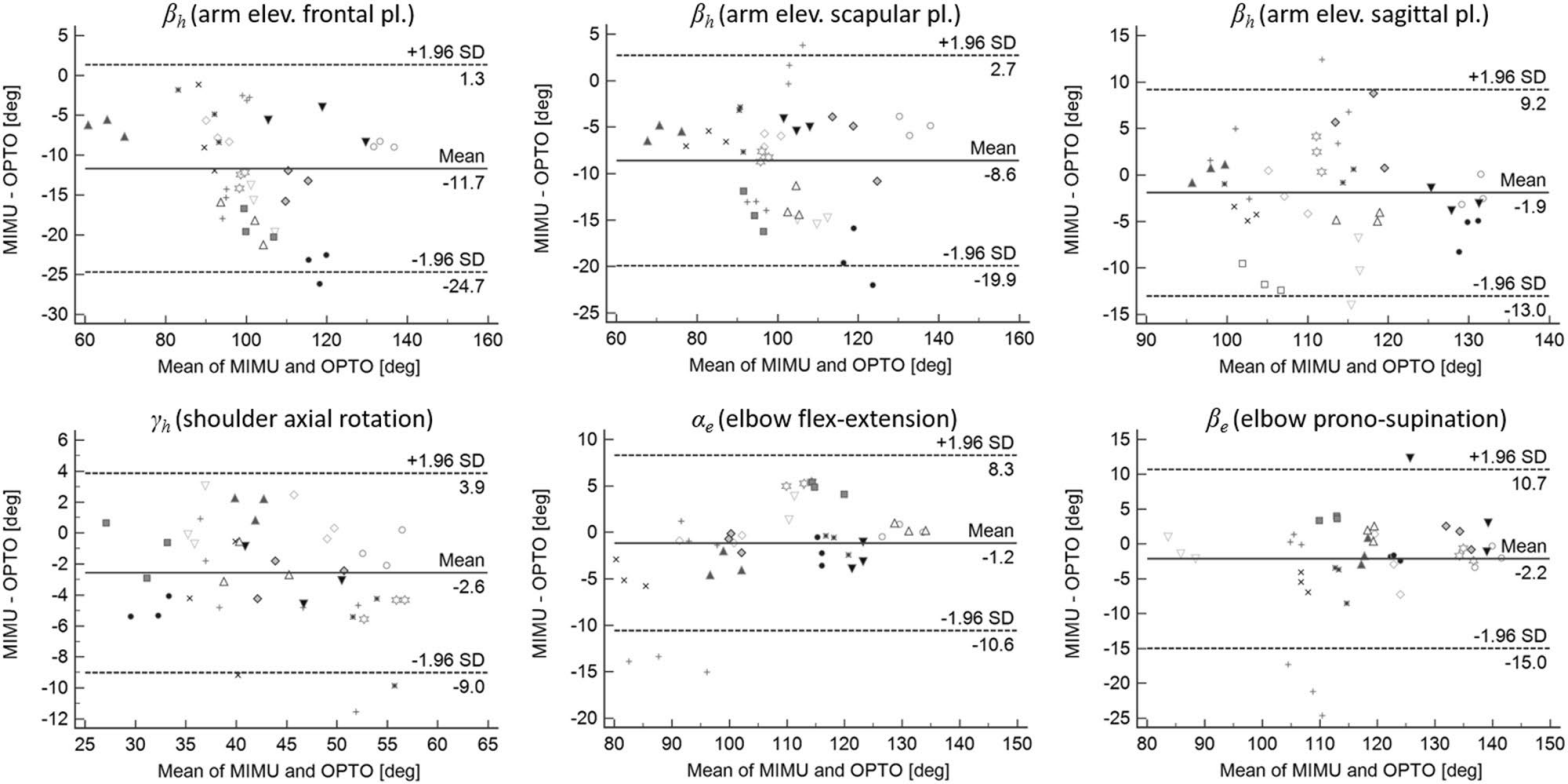
Bland-Altman plots (corrected for the effect of repeated measurements error) showing the agreement between the reference and estimated angular RoM variables considered in the analysis. Bias and 95% upper and lower limits of agreement are reported in the graph. Each point shape is relative to a subject.

RMSD, *R*^2^, *a*_1_ and *a*_0_ values relative to both the angular and linear trajectories comparison were not normally distributed. Hence, these variables are presented in terms of median and inter-quartile range (IQR). Regarding the first experiment, Table 2 reports the reference and estimated angular RoM in addition to the results of the comparison between the reference and estimated angular trajectories. Reference frontal, scapular and sagittal shoulder plane of elevation values (*α*_*h*_) were 8.4° ± 10.8°, 31.8° ± 10.4° and 84° ± 11.8°, respectively, against the corresponding 7.9° ± 12.1°, 33.2° ± 13.4° and 87.2° ± 12.7° estimated by our MIMU-based model. Figure 5 shows the Bland–Altman plots depicting the agreement between the reference and estimated joint angular RoM (bias and 95% lower and upper limits of agreement are reported in the graphs in Fig. 5).

**Table 2:** Mean ± sd of the reference (OPTO) and estimated (MIMU) angular RoM variables considered in the analysis.

Type of motion	Angle	RoM [deg]		RMSD [deg] (% RoM_OPTO_)	*R* ^*2*^	*a*_*1*_	*a*_0_ [deg]
		OPTO	MIMU				
Arm elev. frontal plane	*β*_*h*_	107.1 ± 17.5	95.5 ± 15.6	4.4 ± 4.1 (4.1%)	0.998 ± 0.003	0.921 ± 0.01	−7.8 ± 8.2
Arm elev. scapular plane	*β*_*h*_	105.9 ± 16.4	97.3 ± 15.5	2.5 ± 1.7 (2.2%)	0.997 ± 0.003	0.967 ± 0.06	−2.9 ± 7.5
Arm elev. sagittal plane	*β*_*h*_	114.3 ± 11.4	112.3 ± 10.8	2.3 ± 2.5 (2.2%)	0.997 ± 0.003	1.006 ± 0.05	0.9 ± 5.6
Shoulder axial rotation	*γ*_*h*_	44.9 ± 8.9	42.3 ± 8.1	1.8 ± 1.4 (4.1%)	0.995 ± 0.009	0.951 ± 0.07	−2.0 ± 4
Elbow flex-extension	*α*_*e*_	109.3 ± 13.9	108.1 ± 16.2	1.9 ± 2.6 (1.7%)	0.999 ± 0.002	1.003 ± 0.03	−0.4 ± 4.2
Elbow prono-supination	*β*_*e*_	119.5 ± 14.1	117.3 ± 15.5	2.9 ± 1.6 (2.4%)	0.997 ± 0.004	1.020 ± 0.03	−1.1 ± 1.5

Median ± IQR are reported for RMSD (also expressed as percentage of the reference RoM, RoM_OPTO_) and for the three coefficients obtained from the Linear Fit Method *R*^2^ (coefficient of determination, i.e. waveform similarity), *a*_1_ (slope, i.e. amplitude) and *a*_0_ (intercept, i.e. offset) used for comparing pairs of reference and estimated angular trajectories.

**Figure 6:**
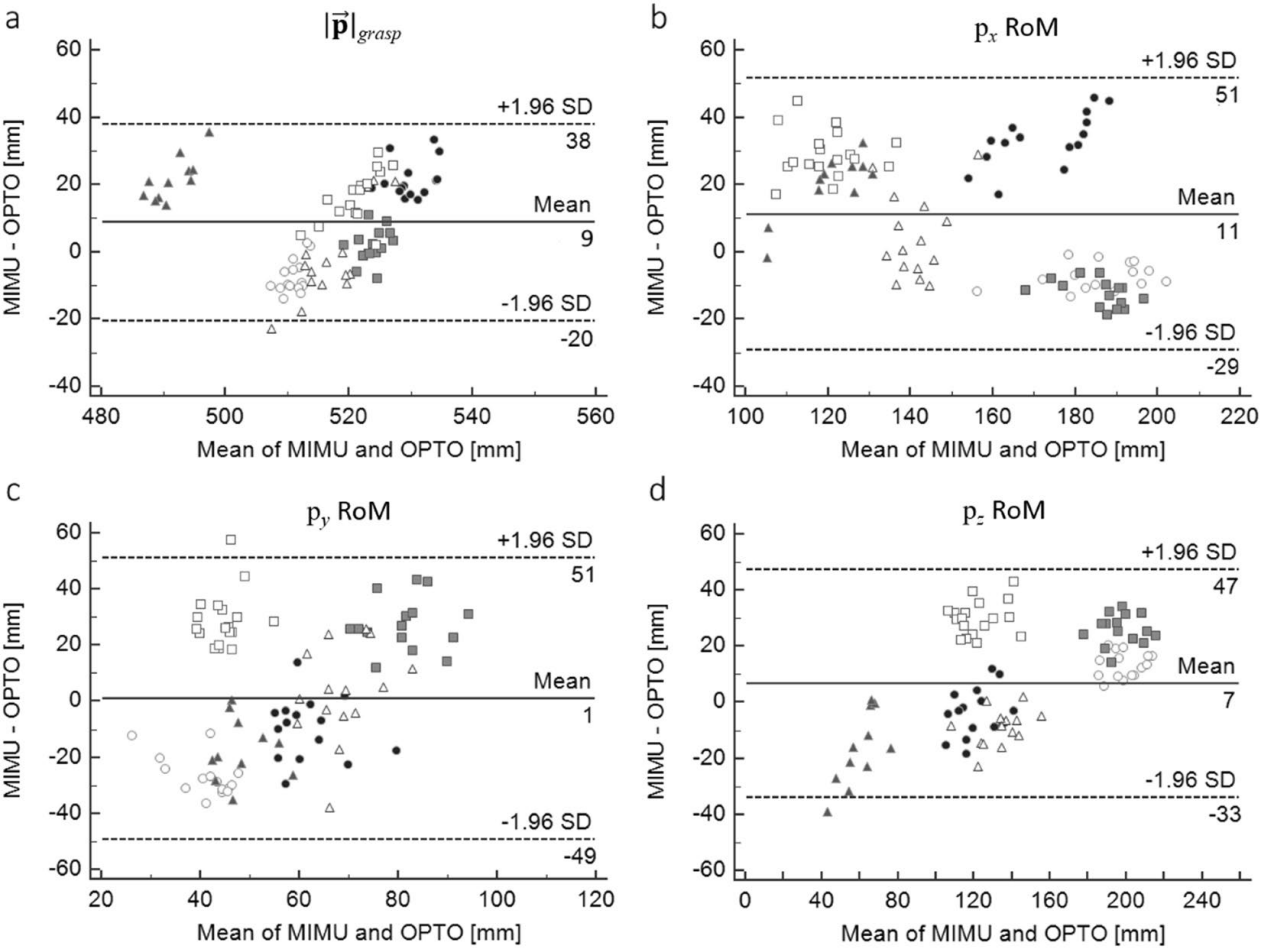
Bland-Altman plots (corrected for the effect of repeated measurements error) showing the agreement between the (a) reference and the estimated magnitude of the ulnar styloid position vector at the grasp point ($|\overrightarrow{{\bf{p}}}|\,$_*grasp*_) and (b-d) between the reference and the estimated linear RoM along the antero-posterior (p_*x*_), longitudinal (p_*y*_) and medio-lateral (p_*z*_) axes of the thorax reference frame. Bias and 95% upper and lower LoAs are reported in the graph. Each point shape is relative to a subject.

Regarding the reach-to-grasp experiment, the target was reached at an average velocity of 0.181 ± 0.06 m/s. Reference and estimated $|\overrightarrow{{\bf{p}}}|$_*grasp*_ values were, on average, 516 ± 13 and 522 ± 14 mm, respectively, and their agreement is reported in Fig. 6a. Heteroscedasticity was observed via visual inspection of the Bland–Altman plot depicted in Fig. 6a and subsequently verified using Kendall’s τ = 0.309. Bias and limits of agreement depicted in Fig. 6a were adjusted accordingly. Finally, Table 3 reports the reference and estimated wrist linear RoM together with the results of the comparison between the reference and estimated linear trajectories; Fig. 6b–d show Bland–Altman plots depicting the agreement between the reference and estimated wrist linear RoM (bias and 95% lower and upper limits of agreement are reported in the graphs in Fig. 6).

**Table 3:** Mean ± sd of reference (OPTO) and estimated (MIMU) linear RoM of the ulnar styloid ([p_*x*_, p_*y*_, p_*z*_]).

	RoM [mm]		RMSD [mm] (% RoM_OPTO_)	*R* ^2^	*a*_1_	*a*_0_ [m]
	OPTO	MIMU				
p_*x*_	149 ± 36	160 ± 26	8 ± 10 (5.1%)	0.968 ± 0.107	0.925 ± 0.228	28 ± 79
p_*y*_	56 ± 16	59 ± 23	15 ± 12 (26.5%)	0.139 ± 0.26	0.229 ± 0.649	−67 ± 93
p_*z*_	138 ± 42	146 ± 53	8 ± 5 (5.6%)	0.977 ± 0.087	1.036 ± 0.224	−1 ± 37

Median ± IQR are reported for RMSD (also expressed as percentage of the reference RoM, RoM_OPTO_) and for the three coefficients obtained from the linear fit method *R*^2^ (coefficient of determination, i.e. waveform similarity), *a*_1_ (slope, i.e. amplitude) and *a*_0_ (intercept, i.e. offset) used for comparing pairs of reference and estimated linear trajectories.

## Discussion

This study proposes an innovative upper limb movement analysis protocol based on the use of wearable MIMUs, which provide anatomically and clinically consistent joint kinematics estimates. The validity of the proposed approach was verified against a camera-based optoelectronic system during two experiments aimed at assessing the accuracy of a two-link open kinematic chain in estimating the 3D shoulder and elbow angular kinematics together with the 3D trajectory of the wrist during standard mobility tests and reach-to-grasp movements.

In the first experiment, the angular RoM were slightly underestimated in a systematic manner, (bias <−2.6°) and they showed a good level of agreement. Largest errors were observed for arm elevation in the frontal plane which was characterised by the highest bias and limits of agreement range (Fig. 5). During the elevation of the arm in the frontal, scapular and sagittal planes, *α*_*h*_ was correctly estimated with <3° of error. When comparing the angular trajectories, the RMSD was <4% of the reference RoM for all considered angular RoMs (Table 2). A high waveform similarity (0.995 < *R*^2^ < 0.999), high amplitude similarity (0.951 < *a*_1_ < 1.02) and small offset (*a*_0_ < 3°) were found for all considered angular variables except for the arm elevation in the frontal plane, which was characterised by the highest offset (Table 2). Unfortunately, none of the previous MIMU-based studies reported the level of accuracy associated with the estimate of upper limb joint kinematics. Assessing the accuracy of the proposed method against a gold standard joint kinematics is, indeed, a highlight of this study as the same anatomical coordinate system determination and same ALs were used both for MIMUs and the reference camera-based motion analysis system.

**Figure 7:**
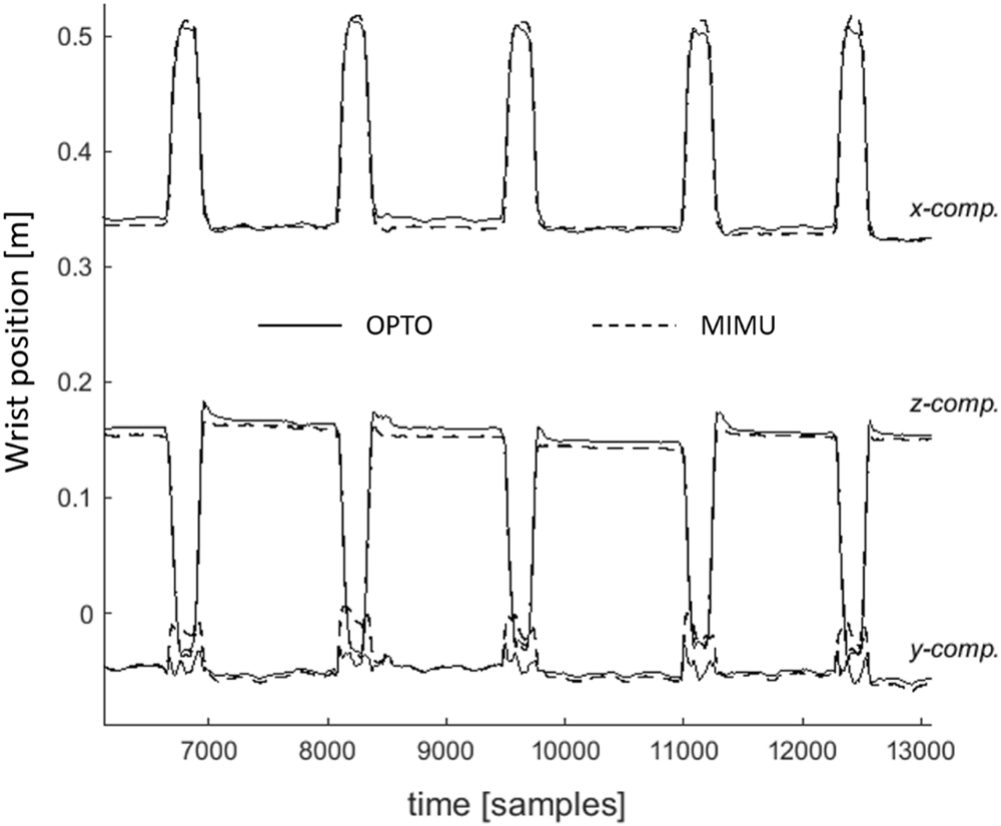
Estimated (solid lines) and reference (dashed lines) three-dimensional components of the instantaneous position of the wrist during five of the fifteen reach-to-grasp cycles of a representative subject.

Regarding the second experiment, the agreement analysis revealed a systematic overestimation of both wrist position and linear RoM (Fig. 6). A low bias (<10 mm) and good level of agreement were found between the reference and estimated $|\overrightarrow{{\bf{p}}}|$_*grasp*_ (Fig. 6a). With regard to the EF trajectory, the best performance was observed in the antero–posterior and medio–lateral directions with a low bias (7 to 11 mm) and small limits of agreement ranges (Fig. 5b,c) in addition to a low RMSD (8 mm, <5.6% of the reference RoM) and offset values, high amplitude and waveform similarity coefficients (Table 3). Conversely, the cranio–caudal direction was characterised by the largest limits of agreement range (Fig. 6c), the largest RMSD and offset values and the lowest waveform and amplitude similarity coefficients (Table 3). An overall picture of the accuracy of the proposed method in tracking the 3D position of the wrist is shown in Fig. 7.

In this study, the magnitude of the errors of the wrist trajectory was comparable with those reported in previous studies based either on double numerical integration with optimisation during reach-to-grasp movements^25^ or on the use of forward kinematics during standard shoulder/elbow mobility tests^26^.

The worst performance in estimating the wrist linear position was observed along the cranio–caudal direction, which was also characterised by the smallest RoM. The source of this error might be found in the task itself, which requires the elevation of the shoulder girdle to allow hand–table clearance. To reduce the number of MIMUs involved, we decided to use a simplified open chain kinematic model of the upper limb that assumes the humerus to be hinged to the thorax and no translational degrees of freedom are considered. Thus, as the scapula is not included in the open chain kinematic model, any translation of the glenohumeral joint (e.g. elevation of the shoulder girdle) would be neglected. The main peculiarity of the proposed method is that it can be applied when dealing with patients presenting severe impaired mobility and/or those equipped with obtrusive medical apparatuses. In such cases, the anatomical landmark identification approach is the most feasible method among those proposed in the literature.

Finally, an important point is that the proposed methodology relies on the assumption that all MIMUs utilised in the protocol share the same global coordinate system, and, therefore, the overall method performance is subject to the quality of the orientation estimates obtained using the sensor fusion algorithm. As this assumption declines in the presence of non-homogenous ferromagnetic disturbances and physical calibration issues of sensors, a system spot-check is necessary prior to data collection^17^.

In conclusion, the proposed anatomical calibration estimated reliable joint angular kinematics in full compliance with the metrological standards of clinical movement analysis. Compared to functional approaches, the proposed anatomical calibration can also be used on bedridden patients who are incapable of performing segment rotations or assuming fixed postures. Additionally, the approach allows one to extend the analysis to linear joint kinematics, providing an adequate spatial resolution for clinical assessment purposes. Consequently, the current study lays the methodological foundation to delve further into the reach-to-grasp kinematics in measurement settings wherein the use of traditional motion capture technologies is not viable. From this perspective, the proposed approach could enable the measurement of the rehabilitative progress of stroke patients from the very acute phase (when patients are bedridden in stroke units) to the chronic stage of the disease.

## Supplementary information

Appendix

## Data Availability

Upon reasonable request, the datasets used and analysed during the current study will be made available by the corresponding author.
