# Supplementary material for: Upper limb joint kinematics using wearable magnetic and inertial measurement units: an anatomical calibration procedure based on bony landmark identification
Source: Sci Rep. 2019 Oct 8;9:14449. doi: 10.1038/s41598-019-50759-z (PMC6783441; doi:10.1038/s41598-019-50759-z)
Supplement: Supplementary file 1 — Appendix [file 41598_2019_50759_MOESM1_ESM.docx]

### TITLE

Upper limb joint kinematics using wearable magnetic and inertial measurement units: an anatomical calibration procedure based on bony landmark identification

### AUTHORS

Pietro Picerno^1^*, Pietro Caliandro^2^, Chiara Iacovelli^3^, Chiara Simbolotti^3^, Michele Crabolu^4,5^, Danilo Pani^4^, Giuseppe Vannozzi^6^, Giuseppe Reale^7^, Paolo Maria Rossini^7^, Luca Padua^3,7^, Andrea Cereatti^8^

1. School of Sport and Exercise Sciences, “e-Campus” University, Novedrate (CO), Italia ([pietro.picerno@uniecampus.it](mailto:pietro.picerno@uniecampus.it))
2. Unità Operativa Complessa di Neurologia, Fondazione Policlinico Universitario A. Gemelli IRCCS, Roma, Italia
3. IRCCS Fondazione Don Carlo Gnocchi, Milano, Italia
4. Department of Electrical and Electronic Engineering, University of Cagliari, Cagliari, Italia
5. Department of Informatics, Bioengineering, Robotics and System Engineering, University of Genoa, Genova, Italia
6. Department of Movement, Human and Health Sciences, University of Rome “Foro Italico,” Roma, Italia
7. Dipartimento di Scienze dell’invecchiamento, Neurologiche, Ortopediche e della Testa-Collo, Università Cattolica del Sacro Cuore, Roma, Italia
8. University of Sassari, Biomedical Sciences Department, Sassari, Italia

### APPENDIX

#### Anatomical landmark abbreviations

##### Palpable ALs

PX: processus xiphoideus

IJ: incisura jugulars

rAC: right acromion

lAC: left acromion

LE: lateral epicondyle

RS: radius styloid

US: ulnar styloid

##### Internal ALs

HH: humeral head

MidE: the point lying in the middle of the line connecting ME and LE

#### Anatomical coordinate system determination

##### Right forearm

The definition of the right forearm anatomical coordinate system (*aF*) follows the International Society of Biomechanics recommendations:

$${{}^{sF}\text{y}}_{aF}= {}^{sF}\hat{US MidE}$$

${{}^{sF}\text{x}}_{aF}= {{}^{sF}\text{y}}_{aF} \Lambda{}^{sF}\hat{US RS}$ (1)

$${{}^{sF}\text{z}}_{aF}= {{}^{\boldsymbol{sF}}\text{x}}_{aF} \Lambda{{}^{sF}\text{y}}_{aF}$$

${{}^{sF}\text{R}}_{aF}= \left[ {{}^{sF}\text{x}}_{aF}, {{}^{sF}\text{y}}_{aF}, {{}^{sF}\text{z}}_{aF} \right]$,

where the longitudinal axis ${{}^{sF}\text{y}}_{aF}$ represents the direction of the vector from US to MidE as estimated via vector summation:

$\vec{US MidE} =\vec{US RS} + \vec{RS LE} - \vec{MidE LE}$, (2)

which is

$${{}^{sF}\vec{US MidE}}_{x}=\left( {{}^{sF}\hat{US RS}}_{i}\cdot\text{D}_{\text{RS-US}} \right)+ \left( {{}^{sF}\hat{RS LE}}_{i}\cdot\text{D}_{\text{LE-RS}} \right)-\left( {{}^{sF}\hat{LE ME}}_{i}\cdot\frac{\text{D}_{\text{LE-ME}}}{2} \right)$$

$${{}^{sF}\vec{US MidE}}_{y}=\left( {{}^{sF}\hat{US RS}}_{j}\cdot\text{D}_{\text{RS-US}} \right)+ \left( {{}^{sF}\hat{RS LE}}_{j}\cdot\text{D}_{\text{LE-RS}} \right)-\left( {{}^{sF}\hat{LE ME}}_{j}\cdot\frac{\text{D}_{\text{LE-ME}}}{2} \right)$$

${{}^{sF}\vec{US MidE}}_{z}=\left( {{}^{sF}\hat{US RS}}_{k}\cdot\text{D}_{\text{RS-US}} \right)+ \left( {{}^{sF}\hat{RS LE}}_{k}\cdot\text{D}_{\text{LE-RS}} \right)-\left( {{}^{sF}\hat{LE ME}}_{k}\cdot\frac{\text{D}_{\text{LE-ME}}}{2} \right)$,

where ${}^{sF}\hat{RS LE}$, ${}^{sF}\hat{LE ME}$ and ${}^{sF}\hat{US RS}$ represent the directions of the pointed anatomical axes with respect to the MIMU fixed on the forearm, and D_LE-ME_, D_LE-RS_ and D_RS-US_ represent the relevant inter-anatomical landmark distances measured by the instrumented pointing device (Fig. 1) during the calibration procedure, respectively. Finally, *i*, *j* and *k* are the three elements composing the pointed unit vectors.

##### Right upper arm

The definition of the right upper arm anatomical coordinate system (*aU*) follows the International Society of Biomechanics recommendations:

$${{}^{sU}\text{y}}_{aU}= {}^{sU}\hat{MidE HH}$$

${{}^{sU}\text{x}}_{aU}= {{}^{sU}\text{y}}_{aU} \Lambda{}^{sU}\hat{ME LE}$ (3)

$${{}^{sU}\text{z}}_{aU}= {{}^{sU}\text{x}}_{aU} \Lambda{{}^{sU}\text{y}}_{aU}$$

${{}^{sU}\text{R}}_{aU}= \left[ {{}^{sU}\text{x}}_{aU}, {{}^{sU}\text{y}}_{aU}, {{}^{sU}\text{z}}_{aU} \right]$,

where the longitudinal axis ${{}^{sU}\text{y}}_{aU}$ represents the direction of the vector from MidE to HH as estimated via vector summation:

$\vec{MidE HH} = \vec{MidE LE} + \vec{LE rAC} - \vec{HH rAC}$, (4)

which is

$${{}^{sU}\vec{MidE HH}}_{x}=\left( {{}^{sU}\hat{ME LE}}_{i}\cdot\frac{\text{D}_{\text{LE-ME}}}{2} \right)+\left( {{}^{sU}\hat{LE rAC}}_{i}\cdot\text{D}_{\text{LE-rAC}} \right)-\left( {}^{sU}{\text{g}_{i}}\cdot\text{D}_{\text{rAC-HH}} \right)$$

$${{}^{sU}\vec{MidE HH}}_{y}=\left( {{}^{sU}\hat{ME LE}}_{j}\cdot\frac{\text{D}_{\text{LE-ME}}}{2} \right)+\left( {{}^{sU}\hat{LE rAC}}_{j}\cdot\text{D}_{\text{LE-rAC}} \right)-\left( {}^{sU}{\text{g}_{j}}\cdot\text{D}_{\text{rAC-HH}} \right)$$

${{}^{sU}\vec{MidE HH}}_{z}=\left( {{}^{sU}\hat{ME LE}}_{k}\cdot\frac{\text{D}_{\text{LE-ME}}}{2} \right)+\left( {{}^{sU}\hat{LE rAC}}_{k}\cdot\text{D}_{\text{LE-rAC}} \right)-\left( {}^{sU}{\text{g}_{k}}\cdot\text{D}_{\text{rAC-HH}} \right)$,

where ${}^{sU}\hat{ME LE}$ and ${}^{sU}\hat{LE rAC}$ represent the directions of the pointed anatomical axes with respect to the MIMU fixed on the upper arm, and D_LE-ME_ and D_rAC-LE_ represent the relevant inter-anatomical landmark distances measured by the instrumented pointing device during the calibration procedure, respectively. ${}^{sU}\text{g}$ and D_rAC-HH_ represent the direction defined by HH to rAC and the relevant inter-anatomical landmark distance, respectively, with the former being the direction of the local gravity (**g**) vector:

${}^{sU}\text{g}= {{{}^{g}\text{R}}_{sU}(0)}^{-1}\cdot\boldsymbol{[}0 0 1\boldsymbol{]}$**.** (5)

The latter is expressed as the percentage of the inter-acromial distance

$\text{D}_{\text{rAC-HH = }}\text{D}_{\text{rAC-lAC }}\cdot0.17,$ (6)

following the anthropometric assumption proposed by Rab et al.^1^.

##### Thorax

The thorax anatomical coordinate system (*aT*) was defined as follows:

$${{}^{sT}\text{y}}_{aT}= {}^{sT}\hat{PX IJ}$$

${{}^{sT}\text{x}}_{aT}= {}^{sT}\hat{lAC rAC}$ (7)

$${{}^{sT}\text{z}}_{aT}= {{}^{sT}\text{x}}_{aT} \Lambda{{}^{sT}\text{y}}_{aT}$$

${{}^{sT}\text{R}}_{aT}=$ [${{}^{sT}\text{x}}_{aT}, {{}^{sT}\text{y}}_{aT}, {{}^{sT}\text{z}}_{aT}$].

Note that, while this anatomical coordinate system definition is consistent with the International Society of Biomechanics recommendation, herein, the inter-acromial line was considered instead of the line joining the eighth thoracic vertebra (T8) to the seventh cervical vertebra (C7). This was because of two reasons: 1) limited accessibility for pointing C7 and T8 spinous processes (e.g. in bedridden patients) and 2) the fact that the inter-acromial distance was already measured to comply with Rab’s assumption for estimating HH position^1^.

#### Forward kinematics of the model

The time-variant homogeneous transformation matrix (**T**) expressing the pose (i.e. position and orientation) of the upper arm (*aU*) and forearm (*aF*) anatomical coordinate systems with respect to the thorax (*aT*) was defined as follows:

${}^{aT}{\text{T}_{aU}}=\left[ \begin{matrix} {}^{aT}{\text{R}_{aU}} & -{}^{aT}{\text{R}_{aU}}\cdot{}^{aU}{\text{p}_{aT}} \\ \begin{matrix} 0 & \begin{matrix} 0 & 0 \end{matrix} \end{matrix} & 1 \end{matrix} \right]$ (8)

${}^{aU}{\text{T}_{aF}}=\left[ \begin{matrix} {}^{aU}{\text{R}_{aF}} & -{}^{aU}{\text{R}_{aF}}\cdot{}^{aF}{\text{p}_{aU}} \\ \begin{matrix} 0 & \begin{matrix} 0 & 0 \end{matrix} \end{matrix} & 1 \end{matrix} \right]$ (9)

${}^{aT}{\text{T}_{aF}}={}^{aT}{\text{T}_{aU}}\cdot{}^{aU}{\text{T}_{aF}}$, (10)

where

${}^{aU}{\text{p}_{aT}}=[0, \text{L}_{\text{HH-MidE}}, 0]$ (11)

${}^{aF}{\text{p}_{aU}}=[0,\text{L}_{\text{MidE-US}}, 0]$. (12)

$\text{L}_{\text{HH-MidE}}$ and $\text{L}_{\text{MidE-US}}$ can be computed as the magnitude of the vectors estimated in Eq. 2 and Eq. 4, respectively. The kinematic chain is linked with spherical joints in MidE and HH, with the latter also being the origin of the system (Fig. 3). The instantaneous 3D position of US can be retrieved from the fourth column of ${}^{aT}{\text{T}_{aF}}$ (Eq. 10).

**REFERENCES**

1. Rab, G., Petuskey, K. & Bagley, A. A method for determination of upper extremity kinematics. *Gait Posture* **15**, 113–119 (2002).
